# Associations of maternal alcohol and non-prescribed substance use with early child growth

**DOI:** 10.4102/sajpsychiatry.v31i0.2486

**Published:** 2025-06-12

**Authors:** Yukiko Washio, Zugui Zhang, Mona Lisa Baishya, Marilyn T. Lake, Bronwyn Myers, Nadia Hoffman, Elizabeth Goddard, Heather J. Zar, Dan J. Stein, Petal Petersen Williams

**Affiliations:** 1 Women’s Global Health Imperative, RTI International, Media, Pennsylvania, United States; 2 Department of Obstetrics, Gynecology and Reproductive Sciences, Lewis Katz School of Medicine, Temple University, Philadelphia, Pennsylvania, United States; 3 iREACH, Christiana Care, Newark, Delaware, United States; 4 College of Public Health, Temple University, Philadelphia, Pennsylvania, United States; 5 SAMRC Unit on Risk and Resilience in Mental Disorders, Department of Psychiatry and Neuroscience Institute, University of Cape Town, Cape Town, South Africa; 6 enAble Institute, Curtin University, Bentley, Australia; 7 SAMRC Unit on Child and Adolescent Health, Department of Pediatrics, University of Cape Town, Cape Town, South Africa; 8 Mental Health, Alcohol, Substance Use and Tobacco Research Unit, South African Medical Research Council, Cape Town, South Africa; 9 Institute for Life Course Health Research, Department of Global Health, Stellenbosch University, South Africa

**Keywords:** pregnancy, early postpartum, infant child length, infant child weight, combined alcohol and substance use

## Abstract

**Background:**

Perinatal alcohol and non-prescribed substance use may be detrimental to foetal and infant growth.

**Aim:**

This observational study investigated how combined and continued alcohol and non-prescribed substance use throughout antenatal and 1-year postnatal periods were associated with adverse child length and weight outcomes up to 24 months.

**Setting:**

Data from participants (*n* = 1098) with information on alcohol and non-prescribed substance use and infant and child outcomes, were drawn from a prospective birth cohort in the Drakenstein Child Health Study (DCHS), conducted in the Western Cape province of South Africa.

**Methods:**

Generalised estimating equations were conducted on standardised child length and weight outcomes at 12, 18 and 24 months.

**Results:**

Non-prescribed substances consisted mostly of tobacco use (77%). Child length and weight were significantly lower in those exposed to the combined use of alcohol and substances compared to no-use and all other use groups (*p* < 0.001), as confirmed by multivariable analyses. Child length and weight were also significantly lower in those exposed to alcohol and/or substance use throughout the antenatal and 1-year postnatal periods, as confirmed by multivariable analyses.

**Conclusion:**

Interventions to address the potential long-term adverse effects of combined alcohol and substance use particularly tobacco use, as well as continuous use throughout antenatal and early postnatal periods on subsequent child growth, are needed.

**Contribution:**

This study has contributed to the field by showing that combined and continued use of alcohol and other substances during pregnancy and postpartum is associated with impaired early child growth.

## Introduction

Antenatal alcohol use is associated with adverse pregnancy, birth and child outcomes, including miscarriage, stillbirth, preterm birth, low birth weight, sudden infant death syndrome and development of foetal alcohol spectrum disorders (FASD).^1,2^ South Africa has the highest FASD rate in the world, followed by Eastern European regions, the United Kingdom and New Zealand.^3,4^ Notably, the Western Cape region of South Africa has detected 20% of biochemically verified antenatal alcohol use,^5^ comparable to the rate of 26% self-reported antenatal alcohol use risk among those receiving services from the Special Supplemental Nutrition Programme for Women, Infants and Children (WIC) in the United States of America (USA).^6^

Combined use of alcohol and other substances, including tobacco, cannabis and methamphetamine, is associated with adverse birth outcomes, including preterm birth, low birth and infant weight and smaller head circumference, compared to antenatal alcohol exposure alone.^7,8,9^ Alcohol use during lactation is associated with antenatal alcohol use and has been shown to adversely impact infant development, including lower verbal IQ scores and reduced infant weight, independently of antenatal alcohol use.^10^ In South Africa, antenatal alcohol and substance use is associated with intimate partner violence, lower socioeconomic status, lack of social support, trauma exposure and depression,^11,12,13^ which in turn may also impact postpartum parenting, thus also impacting infant and child growth.^14,15^

It is not clear whether the combined use of alcohol with any type of non-prescribed substance(s) as well as the continued use of alcohol and/or substances throughout antenatal and postnatal periods is associated with adverse infant and child growth. The aim of this observational study was to investigate the association of perinatal alcohol and non-prescribed substance use with child height and weight through the first 2 years of life. Substances in the analysis of this observational study were non-prescribed substances with a majority being tobacco but also including methamphetamine, cocaine, MDMA, cannabis, opiates and mandrax.

## Research methods and design

### Study design and participants

Data were derived from a prospective birth cohort study of mother–child pairs enrolled in the Drakenstein Child Health Study (DCHS), conducted in a peri-urban area, in the Western Cape province, South Africa. The dataset includes antenatal and postnatal use of alcohol and substance use and child growth standardised outcomes.^16,17^ The DCHS included women during 20–28 weeks of gestation, who were enrolled between March 2012 and March 2015, and subsequently followed from birth as mother–child dyads. Pregnant women were recruited from public primary health care clinics, and all births occurred at a single public hospital serving the area. Pregnant women were eligible to participate in the parent study if they were 18 years and older, accessing antenatal care between 20 and 28 gestational weeks, and intended to remain in the area for at least a year.

Enrolled participants in the study completed interviewer administered self-report questionnaires in their preferred language of English, Afrikaans or isiXhosa (the three local languages of the Western Cape) assessing maternal psychosocial risk factors at antenatal baseline and postnatal time points and child length and weight at 12, 18 and 24 months.^16^ All child assessments were carried out in a private room in one of the study offices; where possible, interviews with the maternal participants were carried out in a separate room or private area. Participants were contacted telephonically or by home visit to schedule study visits.

### Explanatory variables and outcomes

For antenatal (i.e. second trimester), 6-month postpartum and 12-month postpartum alcohol and substance use, ASSIST^18^ questions on use in the past 3 months were used, and a dichotomous variable of use versus non-use at any frequency was created. Urinalysis was conducted for tobacco, methamphetamine, cocaine, MDMA, cannabis, opiates and mandrax at the antenatal baseline visit. For antenatal non-prescribed substance use, a combination of urinalysis results and self-reported use in the past 3 months was used to determine antenatal use, as represented using a dichotomous variable. The variable was categorised as negative only when both urinalysis and self-report indicated negative substance use; otherwise, the variable was categorised as positive of use if either urinalysis or self-report was positive of use. For postnatal use, either 6-month or 12-month positive use was counted as postnatal use at some point during the 1-year postnatal period. Lifetime alcohol and substance use was also assessed to confirm no lifetime use for the no-use groups.

Participants were grouped into four groups based on the type of alcohol and substance use: (1a) no use, (1b) alcohol use only, (1c) substance use only or (1d) alcohol and substance use. Participants were also grouped into four groups based on the timing of alcohol and/or substance use: (2a) no use, (2b) antenatal use only, (2c) postnatal use only or (2d) antenatal and postnatal use. The second grouping was not specific to the type of alcohol or substance use, and the use of any alcohol or substance use was included.

Outcomes included in this analysis were standardised child length and weight scores at 12, 18 and 24 months as continuous variables.^19,20^ Infants are defined as individuals less than 1-year-old and children as 1 year or older.^21^ The infant and child’s length was measured in centimetres to the nearest completed 0.5 cm, using a Seca length-measuring mat (Seca, Hamburg, Germany) for recumbent length up to 18 months and a wall-mounted stadiometer for standing length at 24 months. Infant and child weight was measured in kilograms to the nearest 10 g, in light or no clothing, using the Tanita digital platform scale (TAN1584; IL, USA). Child weight and length measurements were converted to standardised *z*-scores, adjusted for infant age, sex and prematurity. The Fenton growth chart was applied to anthropometry measurements from premature infants (< 37 weeks gestation) at birth and up to 50 weeks postmenstrual age. The anthro software of the World Health Organization was used to adjust for age and sex in measurements taken from full-term infants at birth up to 2 years, as well as age-corrected measurements taken from premature children captured between 50 weeks postmenstrual age and 2 years.^22^ Stunting was defined as the children with length/height-for-age *Z*-score of <−2SD with severe stunting defined as the score of <−3SD.^23^

Sociodemographic variables comprised maternal age, maternal education, marital status, household income, parity, HIV status, maternal height and weight, maternal trauma exposure, maternal intimate partner emotional, physical and sexual violence exposure,^24^ duration of exclusive and any breastfeeding, as well as maternal antenatal depression scores.^25^ The variable of maternal education had two categories of those with less than secondary education level, versus those with at least secondary level education. Household income levels were dichotomised into equal to or less than R5000 and greater than R5000 per month. Marital status was dichotomised into being either single or married/cohabiting. Intimate partner violence exposure variables capturing emotional, physical or sexual abuse in the past 12 months were dichotomized into above and below threshold levels.^26,27^

### Statistical analysis

Descriptive statistics were used to describe the data. Data were summarised using the mean (standard deviations) for continuous variables and using frequency/percentages for categorical variables. A contingency table analysis (Chi-square test) was conducted for comparison of categorical variables, and a non-parametric Kruskal–Wallis test was used to compare continuous variables by each level within each grouping. Statistical significance was set at *p*-value < 0.05.

The generalised estimating equations (GEE) approach was used to model longitudinal outcome measurements of standardised child weight and length at 12, 18 and 24 months against antenatal and postnatal alcohol and/or substance use in the first year of life. Adjusted covariates from participant characteristics included sociodemographic variables as well as those that were statistically significant by univariate comparisons. We included standardised birth height for child height measures and standardised birth weight for child weight measures. Additionally, unadjusted GEE analyses were conducted without covariates, except for grouping and time variables. All analyses were performed using SAS version 9.4 (SAS Institute, Cary NC).

### Ethical considerations

Ethical clearance to conduct this study was obtained from the University of Cape Town Faculty of Health Sciences Human Research Ethics Committee (No. 401/2009), Stellenbosch University (No. N12/02/0002[A1]) and the Western Cape Provincial Health Research Committee (No. 2011RP45). The study was conducted in accordance with the Helsinki Declaration as revised in 2013. Written informed consent was obtained from the participants of the parent study.

## Results

### Participant characteristics

Of 1225 participants enrolled in the DCHS at the second trimester of pregnancy, there were 1143 live births. The current analyses included an analytic sample size of 1098 mother-child dyads, where 45 were excluded due to missing data on alcohol and substance use or infant and child outcomes. When looking at (1) substance type groupings: defined into (1a) no use, (1b) alcohol use only, (1c) substance use only or (1d) alcohol and substance use, 590 of 1098 (54%) participants reported no lifetime alcohol or substance use, 158 (14%) reported alcohol use only during pregnancy and/or postpartum, 88 (8%) reported at least one of the substances but no alcohol use during pregnancy and/or postpartum and 262 (24%) reported both alcohol and substance use during pregnancy and/or postpartum. A majority of substances used antenatally comprised of tobacco use (77%), with smaller proportions testing positive for cannabis and methamphetamine use (3%), mandrax use (2%), opioid, MDMA and cocaine use (< 1%). With respect to (2) timing of alcohol and/or substance use, as defined by (2a) no use, (2b) antenatal use only, (2c) postnatal use only or (2d) antenatal and postnatal use, 590 of 1098 (54%) participants reported no use, 91 (8%) reported antenatal use only, 176 (16%) reported postnatal use only and 241 (22%) reported use throughout antenatal and postnatal periods.

Participant characteristics by group comparison are shown in Table 1 and Table 2. The total cohort included in the analyses (*N* = 1098) had children with an average length of 50 centimetres and three kilograms at birth. In Table 1, with grouping by substance use type, substance use only and alcohol and substance use were significantly associated with reduced height measures compared to no use and alcohol use only (*p* < 0.001). Child weight measures showed progressively reduced weight measures in the order of no use, alcohol use only, substance use only and alcohol and substance use with statistically significant differences (*p* < 0.001). In Table 2, with grouping by timing of substance use, all child measures showed statistical differences by group, with the most compromised growth measures associated with the antenatal and postnatal use group compared to no-use, antenatal-only and postnatal-only groups (*p* < 0.001). All groups showed progressively reduced length measures from 12 to 24 months, while all groups showed reduced weight measures at 12 and 24 months compared to 18 months (Table 1 and Table 2).

**TABLE 1:** Participant characteristics by substance use type (*N* = 1098).

Variable	Total (*N* = 1098)	s.d.	%	No use (*n* = 590)	s.d.	%	Alcohol use only (*n* = 158)	s.d.	%	Substance use only (*n* = 88)	s.d.	%	Alcohol and substance use (*n* = 262)	s.d.	%	*p*
Maternal age at enrollment, mean	26.63	5.72	-	27.28	5.75	-	26.45	5.99	-	25.54	5.54	-	25.62	5.35	-	< 0.001
Lower than secondary education	61.58	-	676/1098	57.97	-	342/590	60.13	-	95/158	72.73	-	64/88	66.79	-	175/262	0.012
Household income R5000/month or less	86.61	-	951/1098	86.61	-	511/590	83.54	-	132/158	89.77	-	79/88	87.40	-	229/262	ns
Married or cohabiting	41.29	-	453/1098	43.97	-	259/590	27.85	-	44/158	46.59	-	41/88	41.60	-	109/262	0.002
Antenatal maternal height, mean	159.41	6.79	-	160.07	6.87	-	158.75	6.80	-	157.95	5.35	-	158.85	6.92	-	0.005
Parity, mean	1.04	1.05	-	1.06	1.01	-	1.00	1.10	-	1.13	1.22	-	0.97	1.07	-	ns
HIV-positive status	22.04	-	242/1098	31.02	-	183/590	18.35	-	29/158	9.09	-	8/88	8.40	-	22/262	< 0.001
Depression scale score antenatal, mean	9.45	5.28	-	9.52	4.70	-	9.19	5.29	-	8.49	5.59	-	9.79	6.24	-	ns
Exposed to traumatic events	25.63	-	255/1098	29.23	-	152/590	22.15	-	33/158	21.69	-	18/88	21.40	-	52/262	ns
Past 12 months emotional abuse	26.69	-	265/1098	16.25	-	84/590	32.00	-	48/158	33.73	-	28/88	43.21	-	105/262	< 0.001
Past 12 months physical abuse	21.85	-	217/1098	16.05	-	83/590	22.00	-	33/158	26.51	-	22/88	32.51	-	79/262	< 0.001
Past 12 months sexual abuse	6.85	-	68/1098	3.68	-	19/590	8.00	-	12/158	10.84	-	9/88	11.52	-	28/262	< 0.001
Infant length at birth (cm), mean	49.64	3.73	-	50.01	3.68	-	49.98	3.52	-	49.30	3.48	-	48.76	3.91	-	< 0.001
Standardised infant length at 12 months, mean	−0.63	1.26	-	−0.38	1.16	-	−0.69	1.34	-	−0.96	1.35	-	−0.92	1.28	-	< 0.001
Standardised child length at 18 months, mean	−0.88	1.45	-	−0.73	1.34	-	−0.95	1.54	-	−0.96	1.55	-	−1.12	1.52	-	< 0.001
Standardised child length at 24 months, mean	−1.06	1.20	-	−0.88	1.15	-	−1.04	1.33	-	−1.41	1.31	-	−1.29	1.12	-	< 0.001
Infant weight at birth (kg), mean	3.02	0.60	-	3.08	0.59	-	3.09	0.55	-	2.97	0.60	-	2.87	0.63	-	< 0.001
Standardised infant weight at 12 months, mean	−0.02	1.29	-	0.28	1.28	-	−0.06	1.30	-	−0.32	1.15	-	−0.46	1.21	-	< 0.001
Standardised child weight at 18 months, mean	0.11	1.32	-	0.40	1.30	-	−0.03	1.33	-	−0.13	1.37	-	−0.29	1.22	-	< 0.001
Standardised child weight at 24 months, mean	−0.29	1.23	-	−0.00	1.21	-	−0.37	1.23	-	−0.50	1.20	-	−0.68	1.15	-	< 0.001
# months exclusive breastfeeding, mean	2.09	1.96	-	2.01	2.01	-	1.96	1.94	-	2.11	1.94	-	2.32	1.87	-	0.008
# months any breastfeeding, mean	9.91	9.50	-	7.80	8.31	-	9.35	9.65	-	12.46	10.58	-	14.02	9.98	-	< 0.001

s.d., standard deviation; ns, not significant.

**TABLE 2:** Participant characteristics by when alcohol and substance were used (*N* = 1098).

Variable	Total (*N* = 1098)	s.d.	%	No use (*n* = 590)	s.d.	%	Antenatal only (*n* = 91)	s.d.	%	Postnatal only (*n* = 176)	s.d.	%	Antenatal and postnatal (*n* = 241)	s.d.	%	*p*
Maternal age at enrollment, mean	26.63	5.72	-	27.28	5.75	-	25.86	5.20	-	26.03	6.02	-	25.74	5.42	-	< 0.001
Lower than secondary education	61.58	-	676/1098	57.97	-	342/590	70.33	-	64/91	56.25	-	99/176	70.95	-	171/241	< 0.001
Household income R5000/mos or less	86.61	-	951/1098	86.61	-	511/590	90.11	-	82/91	82.39	-	145/176	88.38	-	213/241	ns
Married or cohabiting	41.29	-	453/1098	43.97	-	259/590	43.96	-	40/91	30.11	-	53/176	41.91	-	101/241	0.011
Antenatal maternal height, mean	159.41	6.79	-	160.07	6.87	-	158.97	5.12	-	158.60	6.98	-	158.59	6.89	-	0.006
Parity, mean	1.04	1.05	-	1.06	1.01	-	1.07	1.11	-	0.94	1.13	-	1.03	1.09	-	ns
HIV-positive status	22.04	-	242/1098	31.02	-	183/590	19.78	-	18/91	13.07	-	23/176	7.47	-	18/241	< 0.001
Depression scale score antenatal, mean	9.45	5.28	-	9.52	4.70	-	9.56	5.88	-	8.65	5.26	-	9.74	6.15	-	ns
Exposed to traumatic events	25.63	-	255/1098	29.23	152/590	-	24.18	-	22/91	20.28	-	29/176	21.58	-	52/241	0.049
Past 12 months emotional abuse	26.69	-	265/1098	16.25	84/590	-	42.86	-	39/91	27.78	-	40/176	42.32	-	102/241	< 0.001
Past 12 months physical abuse	21.85	-	217/1098	16.05	83/590	-	32.97	-	30/91	18.75	-	27/176	31.95	-	77/241	< 0.001
Past 12 months sexual abuse	6.85	-	68/1098	3.68	19/590	-	13.19	-	12/91	6.25	-	9/176	11.62	-	28/241	< 0.001
Infant length at birth (cm), mean	49.64	3.73	-	50.01	3.68	-	48.79	3.48	-	50.20	3.45	-	48.70	3.93	-	< 0.001
Standardised infant length at 12 months, mean	−0.63	1.26	-	−0.38	1.16	-	−0.71	1.46	-	−0.69	1.26	-	−1.00	1.30	-	< 0.001
Standardised child length at 18 months, mean	−0.88	1.45	-	−0.73	1.34	-	−0.89	1.50	-	−0.90	1.53	-	−1.16	1.54	-	< 0.001
Standardised child length at 24 months, mean	−1.06	1.20	-	−0.88	1.15	-	−1.25	1.42	-	−1.03	1.26	-	−1.35	1.14	-	< 0.001
Infant weight at birth (kg), mean	3.02	0.60	-	3.08	0.59	-	2.95	0.59	-	3.12	0.58	-	2.84	0.61	-	< 0.001
Standardised infant weight at 12 months, mean	−0.02	1.29	-	0.28	1.28	-	−0.16	1.06	-	−0.08	1.26	-	−0.50	1.24	-	< 0.001
Standardised child weight at 18 months, mean	0.11	1.32	-	0.40	1.30	-	−0.13	1.26	-	0.03	1.30	-	−0.33	1.25	-	< 0.001
Standardised child weight at 24 months, mean	−0.29	1.23	-	−0.00	1.21	-	−0.40	1.16	-	−0.33	1.22	-	−0.75	1.15	-	< 0.001
# months exclusive breastfeeding, mean	2.09	1.96	-	2.01	2.01	-	1.59	1.72	-	2.21	1.92	-	2.33	1.93	-	0.002
# months any breastfeeding, mean	9.91	9.50	-	7.80	8.31	-	7.90	9.51	-	10.49	9.51	-	14.99	10.09	-	< 0.001

s.d., standard deviation; ns, not significant.

In the cohort included in the analyses (*N* = 1098), participants were 27 years old on average with more than half having lower than secondary education, 87% earning R5000 per month or less as the household income, 41% married or cohabiting and 22% HIV seropositive. Approximately 25% were exposed to a traumatic event with 27% suffering from emotional abuse (Table 1 and Table 2). In the first grouping by substance use type, the no-use group was older than the other use groups and had higher levels of education (Table 1). A much higher rate of HIV seropositivity was found in the no-use group compared to the other use groups. Depression scores were similar across all groups. Progressively higher rates of past year emotional, physical and sexual abuse were observed in the order of no use, alcohol use only, substance use only and alcohol and substance use. In the second grouping by the timing of substance use, the no-use group was older and had a higher rate of HIV seropositivity than the other use groups (Table 2). Depression scores were similar across all groups. Higher rates of emotional, physical and sexual abuse were observed among antenatal-only and antenatal and postnatal use groups compared to no-use and postnatal-only groups.

### Child length and weight associated with substance use type

Table 3 shows the multivariable analyses by alcohol and substance use type with adjusted estimates on the standardised child length and weight as repeated measures at 12, 18 and 24 months. Alcohol use only was not significantly associated with shorter child length but there was a statistically significant reduction in child weight compared to the no-use group (*p* = 0.516 and *p* = 0.019). Substance use only was not significantly associated with shorter child length but there was a statistically significant reduction in child weight compared to the no-use group (*p* = 0.094 and *p* = 0.022). Alcohol and substance combined use was significantly associated with reduced child length and weight compared to the no-use group (*p* = 0.002 and *p* < 0.001). Findings from unadjusted GEE analyses mostly confirmed the findings of the multivariable analyses (see Supplementary Table 1).

**TABLE 3:** Child length and weight associated with substance use type (*N* = 1098).

Variables	Estimate	s.e.	95% CI		*P*
**Child length**					
Intercept	−10.91	0.92	−12.71	−9.11	< 0.01
**Substance use type (Ref: No use)**					
Alcohol use only	−0.07	0.11	−0.28	0.14	ns
Substance use only	−0.25	0.15	−0.54	0.04	ns
Alcohol and any other substance use	−0.28	0.09	−0.45	−0.10	< 0.01
Months (12, 18 and 24 months)	−0.04	< 0.01	−0.04	−0.03	< 0.01
Maternal age	−0.01	0.01	−0.02	0.01	ns
Maternal education less than secondary (Ref: at least secondary education)	−0.31	0.07	−0.45	−0.17	< 0.01
Marital status not married/cohabiting (Ref: Married/cohabiting)	−0.06	0.08	−0.21	0.10	ns
Antenatal maternal height	0.04	0.01	0.03	0.05	< 0.01
Maternal HIV positive (Ref: negative)	−0.23	0.10	−0.43	−0.03	0.02
Trauma exposure (Ref: not exposed)	0.10	0.08	−0.05	0.26	ns
Past 12 months emotional abuse exposure (Ref: not exposed)	0.09	0.10	−0.11	0.28	ns
Past 12 months physical abuse exposure (Ref: not exposed)	< 0.01	0.10	−0.20	0.20	ns
Past 12 months sexual abuse exposure (Ref: not exposed)	−0.01	0.16	−0.32	0.31	ns
Infant length at birth (cm)	0.09	0.01	0.07	0.11	< 0.01
# months exclusive breastfeeding	−0.03	0.02	−0.06	0.01	ns
# months any breastfeeding	−0.01	0.01	−0.02	< 0.01	0.01
**Child weight**					
Intercept	−5.67	0.90	−7.43	−3.90	< 0.01
**Substance use type (Ref: No use)**					
Alcohol use only	−0.24	0.10	−0.44	−0.04	0.02
Substance use only	−0.32	0.14	−0.59	−0.05	0.02
Alcohol and any other substance use	−0.43	0.09	−0.61	−0.24	< 0.01
Months (12, 18 and 24 months)	−0.02	< 0.01	−0.02	−0.01	< 0.01
Maternal age	< 0.01	0.01	−0.01	0.02	ns
Maternal education less than secondary (Ref: at least secondary education)	−0.33	0.08	−0.48	−0.18	< 0.01
Marital status not married/cohabiting (Ref: married/cohabiting)	0.02	0.08	−0.14	0.18	ns
Antenatal maternal height	0.03	0.01	0.02	0.04	< 0.01
Maternal HIV positive (Ref: negative)	−0.18	0.11	−0.39	0.03	ns
Trauma exposure (Ref: not exposed)	0.05	0.08	−0.12	0.22	ns
Past 12 months emotional abuse exposure (Ref: not exposed)	0.13	0.10	−0.08	0.34	ns
Past 12 months physical abuse exposure (Ref: not exposed)	< 0.01	0.11	−0.21	0.21	ns
Past 12 months sexual abuse exposure (Ref: not exposed)	−0.22	0.15	−0.52	0.09	ns
Infant weight at birth (kg)	0.72	0.07	0.58	0.86	< 0.01
# months exclusive breastfeeding	−0.02	0.02	−0.06	0.01	ns
# months any breastfeeding	−0.02	< 0.01	−0.03	−0.02	< 0.01

CI, confidence interval; s.e., standard error; ns, not significant; Ref, reference.

### Child length and weight associated with use timing

Table 4 shows the multivariable analyses by when use occurred with adjusted estimates on the standardised child length and weight as repeated measures at 12, 18 and 24 months. Antenatal use only and postnatal use only were not significantly associated with reduced child length compared to the no-use group (*p* = 0.374 and *p* = 0.219); however, use throughout antenatal and postnatal periods showed a significant association with reduced child length compared to the no-use group (*p* = 0.004). Reduced child weight, on the other hand, was significantly associated with antenatal use only, postnatal use only and use throughout antenatal and postnatal periods compared to no use (*p* = 0.019, *p* = 0.009 and *p* < 0.001). Findings from unadjusted GEE analyses confirmed the findings of the multivariable analyses (see Supplementary Table 2).

**TABLE 4:** Child length and weight associated with period of use (*N* = 1098).

Variables	Estimate	s.e.	95% CI		*P*
**Child length**					
Intercept	−10.88	0.92	−12.67	−9.08	< 0.01
**Substance use timing (Ref: No use)**					
Antenatal use only	−0.14	0.16	−0.45	0.17	ns
Postpartum use only	−0.13	0.11	−0.34	0.08	ns
Use throughout pregnancy and postpartum periods	−0.26	0.09	−0.44	−0.09	< 0.01
Months (12, 18 and 24 months)	−0.04	< 0.01	−0.04	−0.03	< 0.01
Maternal age	−0.01	0.01	−0.02	0.01	ns
Maternal education less than secondary (Ref: at least secondary education)	−0.31	0.07	−0.45	−0.16	< 0.01
Marital status not married/cohabiting (Ref: married/cohabiting)	−0.05	0.08	−0.20	0.11	ns
Antenatal maternal height	0.04	0.01	0.03	0.05	< 0.01
Maternal HIV positive (Ref: negative)	−0.23	0.10	−0.43	−0.03	0.03
Trauma exposure (Ref: not exposed)	0.11	0.08	−0.05	0.26	ns
Past 12 months emotional abuse exposure (Ref: not exposed)	0.09	0.10	−0.11	0.28	ns
Past 12 months physical abuse exposure (Ref: not exposed)	< 0.01	0.10	−0.20	0.20	ns
Past 12 months sexual abuse exposure (Ref: not exposed)	< 0.01	0.16	−0.32	0.31	ns
Infant length at birth (cm)	0.09	0.01	0.07	0.11	< 0.01
# months exclusive breastfeeding	−0.03	0.02	−0.07	0.01	ns
# months any breastfeeding	−0.01	0.01	−0.02	< 0.01	0.01
**Child weight**					
Intercept	−5.63	0.90	−7.40	−3.86	< 0.01
**Substance use timing (Ref: No use)**					
Antenatal use only	−0.34	0.15	−0.63	−0.06	0.02
Postpartum use only	−0.27	0.10	−0.47	−0.07	0.01
Use throughout pregnancy and postpartum periods	−0.40	0.09	−0.58	−0.21	< 0.01
Months (12, 18 and 24 months)	−0.02	< 0.01	−0.02	−0.01	< 0.01
Maternal age	< 0.01	0.01	−0.01	0.02	ns
Maternal education less than secondary (Ref: at least secondary education)	−0.32	0.08	−0.47	−0.17	< 0.01
Marital status not married/cohabiting (Ref: married/cohabiting)	0.03	0.08	−0.13	0.18	ns
Antenatal maternal height	0.03	0.01	0.02	0.04	< 0.01
Maternal HIV positive (Ref: negative)	−0.17	0.11	−0.38	0.03	ns
Trauma exposure (Ref: not exposed)	0.05	0.08	−0.11	0.22	ns
Past 12 months emotional abuse exposure (Ref: not exposed)	0.13	0.11	−0.08	0.34	ns
Past 12 months physical abuse exposure (Ref: not exposed)	< 0.01	0.11	−0.21	0.20	ns
Past 12 months sexual abuse exposure (Ref: not exposed)	−0.21	0.15	−0.51	0.09	ns
Infant weight at birth (kg)	0.72	0.07	0.58	0.86	< 0.01
# months exclusive breastfeeding	−0.02	0.02	−0.06	0.01	ns
# months any breastfeeding	−0.02	< 0.01	−0.03	−0.02	< 0.01

CI, confidence interval; s.e., standard error; ns, not significant; Ref, reference.

### Child length and weight associated with other covariates

Statistically significant covariates besides the grouping variables on the standardised child length and weight included months (i.e. 12, 18 and 24), maternal education and height, birth length or weight and any breastfeeding duration (Table 3 and Table 4). Maternal education and height, as well as birth length or weight, were positively associated with child length and weight, indicating that higher maternal height and education and greater birth length or weight were associated with increased child length and weight. Longer duration of any breastfeeding was inversely associated with child length and weight. Maternal trauma exposure and past 12 month abuse exposure variables did not show any significant associations with child length and weight.

## Discussion

### Key findings

The current study was a secondary analysis of data based on an ongoing prospective study of mother–child pairs enrolled in the DCHS conducted in Western Cape, South Africa. Reduced standardised child length and weight outcomes at 12, 18 and 24 months were significantly associated with combined alcohol and non-prescribed substance use and continued use throughout antenatal and 1-year postnatal periods compared to no use. Alcohol use-only, substance use-only, antenatal use-only and postnatal use-only groups were significantly associated with reduced infant and child weight compared to no use, however, not with infant and child length. Multivariable models showed that time (i.e. 12, 18 and 24 months), maternal height and education, birth length or weight, and breastfeeding duration were significantly associated with child outcomes.

### Discussion of key findings

None of the average standardised stunting scores across the groups was within the range of severe stunting. The current report provided evidence that both combined use of alcohol and non-prescribed substances and continued use throughout antenatal and 1-year postnatal periods were significantly associated with reduced child length and weight outcomes up to 24 months, compared to no use. Notably, child weight was significantly less in the alcohol use-only group and in the antenatal-only and postnatal-only groups, compared to the no-use groups. A majority of non-prescribed substances used comprised tobacco, which adversely impacts infant birth weight^28^; thus, the weight outcomes reflected the high prevalence of tobacco use in the samples. Combined use of alcohol and tobacco worsens birth outcomes, including infant weight compared to antenatal alcohol exposure alone.^9^ The current study expands the previous evidence with findings that combined use showed more compromised growth up to 24 months of life compared to alcohol use alone.

The current report showed that a longer duration of breastfeeding was independently significantly associated with decreased child length and weight outcomes, and having at least a secondary level of education was associated with positive child length and weight outcomes. These findings were consistent with the existing body of literature on food insecurity’s impact on breastfeeding in underserved communities^29^ and social determinants of health on child growth.^30,31,32^

Although associations of maternal trauma and past 12-month interpersonal violence with child length and weight were not significant, addressing maternal substance and alcohol use as well as relevant contextual issues such as violence and trauma exposure as a syndemic is important.

## Strengths and limitations

The current report contributes to the field of maternal alcohol and non-prescribed substance use and infant and child outcomes, showing that combined use of alcohol and non-prescribed substances was associated with the most compromised child length and weight outcomes up to 24 months. The current report also added to the body of the evidence on perinatal alcohol and/or non-prescribed substance use with the evidence that use throughout antenatal and 1-year postnatal periods was also associated with the most compromised child length and weight outcomes up to 24 months.

Several limitations of the current report deserve emphasis. Firstly, some of the responses to the alcohol and substance use questions were missing, which lowered the sample size. Secondly, alcohol use was addressed only by self-report without biochemical verification although ASSIST questions on the past 3-month use are well validated across multiple populations and settings.^33^ Substance use during postpartum was also only by self-report without biochemical verification. Thirdly, multiple substance use, without a sufficient number of participants using each, could not be dismantled to investigate the independent adverse effects of each substance use on child growth. Fourthly, dietary diversity was not investigated in this study cohort. Fifthly, this was an observational study and could not investigate a causal relationship between variables and child growth beyond associations.

## Conclusion

The current study demonstrated the adverse associations of maternal alcohol and non-prescribed substance use with early child growth. Most of the non-prescribed substance comprised tobacco use, and combined adverse effects might have resulted from alcohol and tobacco co-use. Much emphasis on addressing combined and continued use of alcohol and substances during pregnancy and early postpartum needs to be made in terms of the potential long-term synergetic adverse effects on subsequent child growth. Proper education, guidance and intervention to reduce antenatal and postnatal combined use of alcohol and substances are urgently needed for optimal child length and weight outcomes. Other associated mental health and violence-related issues should also be addressed as contextual issues.
